# Does school SES matter less for high-performing students than for their lower-performing peers? A quantile regression analysis of PISA 2018 Australia

**DOI:** 10.1186/s40536-022-00137-5

**Published:** 2022-11-11

**Authors:** Laura B. Perry, Argun Saatcioglu, Roslyn Arlin Mickelson

**Affiliations:** 1 School of Education, Murdoch University, Perth, Australia; 2 School of Education and Human Sciences, University of Kansas, Lawrence, USA; 3 Department of Sociology, University of North Carolina, Charlotte, USA

## Abstract

**Background:**

While the relationship between school socioeconomic composition and student academic outcomes is well established, knowledge about differential effects is not extensive. In particular, little is known whether the relationship differs for students with varying levels of academic performance. We examined whether the school socioeconomic composition effect on academic achievement is stronger or weaker for high-performing students than for average- and low-performing students. Australia is a theoretically interesting case study as it has high levels of school socioeconomic segregation compared to other economically developed countries.

**Methods:**

We conducted quantile regression analysis using data from the Australia PISA 2018 sample (N = 14,273 15-year-old students). We examined the effect of school socioeconomic status (school SES) on student performance in reading, mathematical and scientific literacy.

**Results:**

We found that the school socioeconomic composition effect is substantial and is similar for all students, regardless of their level of academic performance. The findings also show that school SES is a stronger predictor than student SES for all student performance quintiles, and the size of the school SES effect relative to the size of student SES effect is larger in lower performance quintiles.

**Conclusions:**

These results indicate no differential effect of school SES on reading, mathematical or scientific literacy for students of varying levels of academic performance. The relationship is similarly strong and positive for high-performing students as it is for their lower performing peers. As school SES is a strong predictor for all students regardless of their level of academic performance, we argue that equity of educational outcomes can be best achieved by policies and structures that promote socioeconomically mixed rather than segregated schools. We also call for more research that seeks to identify and understand possible differential effects of school socioeconomic composition on a range of academic and non-cognitive student outcomes.

## Background

Understanding how to reduce educational inequalities is a central concern in educational research. Despite decades of research and reform, however, substantial reductions in educational inequalities have not been achieved. While various school-based programs have been shown to increase the outcomes of low-income and other socially disadvantaged students, consistent and long-term reductions are not sustained nor scalable [taken to scale] (Berliner, 2014; Thomas et al., 2007). This is in large part because school initiatives do not address the underlying structures that cause educational inequalities.

One such structural factor that is associated with educational inequality is the non-random sorting (i.e., segregation) of students by family income and/or socioeconomic status (SES) among schools. SES segregation is typically associated with neighbourhood attendance zones, marketization, and school choice (Lubienski et al., 2022; Perry et al., 2022). When students are sorted among schools resulting in large concentrations of low SES students in some schools and high SES students in other schools, schooling is segregated by socioeconomic status. Socioeconomic segregation between schools is problematic because it is associated with unequal opportunities to learn (Owens, 2018). Schools with high concentrations of low SES/low-income students usually have fewer human and material resources (Akiba et al., 2007; Chiu & Khoo, 2005; Darling-Hammond, 2010) and reduced learning opportunities (Camburn & Han, 2011; Reardon, 2011). Reduced educational opportunities are linked with stunted outcomes. Students who attend schools with high concentrations of low-income peers are not as academically successful—as measured by grades, test scores, promotion, and graduation rates— as their observationally comparable schoolmates who attend more socioeconomically (and racially) diverse schools (Duncan & Murnane, 2011; Owens, 2018; Reardon, 2011; Schwartz, 2010; Wilms, 1986).

Despite decades of research that shows conclusively a relationship between school SES and student outcomes, ***little is known about any differential effects of school socioeconomic composition for students with varying levels of academic performance***. Understanding whether and the degree to which the relationship between school socioeconomic composition effects on academic achievement varies by performance level has important implications for policy and practice as well as the school choice behaviours of families. For example, if school socioeconomic composition effects are minimal for high-performing students, parents of such children may feel less of a need to choose a non-local public school because evidence shows that their high-performing child will be academically successful regardless of the socioeconomic composition of the school. For policymakers, understanding whether school socioeconomic composition effects are differential or not could inform justifications for reducing school socioeconomic segregation. If school socioeconomic composition effects are not differential, then policy arguments would stress the reduction of school socioeconomic segregation as a way to reduce zero sum scenarios where “winners take all” and the Matthew effect, where structures privilege the already privileged, leading to a scenario in which the “rich get richer and the poor get poorer”. By contrast, differential effects that more negatively impact lower achieving students would justify reductions in school socioeconomic segregation as a way to improve overall achievement across the entire education system. Of course, if school socioeconomic composition effects on student achievement are minimal, then school socioeconomic segregation may not be problematic, thereby removing the policy imperative to address it. We note, however, that school socioeconomic segregation could have negative effects on other outcomes, such as social cohesion and tolerance of difference.

The aim of this study is to generate new knowledge about the differential effects of school SES. Specifically, ***our aim is to examine whether the school socioeconomic composition effect varies for Australian students with different levels of academic performance*** on the Organisation for Economic Cooperation and Development (OECD)’s PISA 2018 assessments of mathematics, science, and reading literacy. We also examine if school SES is more or less associated with performance relative to other significant predictors of achievement among academically stronger students compared to their academically weaker peers. To the best of our knowledge, prior studies have not examined these questions.

Inspiration for our study comes from Giambona and Porcu (2015), who examined differential effects of school type and school location for students with varying levels of academic performance in Italy. Our study builds on their work by including school SES as the primary independent variable of interest. Given the rarely disputed contribution of school SES to student outcomes over and above the role of individual characteristics, and the persistence of SES segregation among schools in Australia and most other countries, this study contributes to the corpus of scientific knowledge about academic outcomes and school socioeconomic segregation.

Australia is a theoretically significant case study for examining these questions as it has high levels of school socioeconomic segregation driven in large part by a marketized educational context. Over several decades, public policymaking has promoted school choice and competition, leading to a stratified system of schooling divided by school sector. This stratification manifests with a large private school sector that receives funding from both private sources (tuition fees paid by families) and public sources (state and federal funding to schools). Even high-fee private schools receive public funding, leading to large between-school inequalities of human and material resources (Connors & McMorrow, 2015). Overall, inequalities in human and material resources between socially advantaged and disadvantaged schools (whether public or private) in Australia are among the largest in the Organisation for Economic Cooperation and Development (OECD) (Cobbold, 2017).

Studies of school socioeconomic composition effects in Australia have the potential to provide evidence for informing policy efforts to reduce educational inequalities, a laudable goal given the stratified nature of schooling in Australia. Moreover, examining differential school socioeconomic composition effects in Australia can contribute to the development of a larger theoretical framework about the causes, mechanisms and consequences of school socioeconomic composition effects. Educational policies and contexts typically vary more between countries than within them, so contextually rich studies of individual countries are necessary for developing rigorous theory. Conducting studies of individual countries and their systems of schooling can enable the development of a robust explanatory theoretical framework about the policies, contexts and conditions that influence school socioeconomic composition effects, as well as the policy levers that may be used to mitigate their negative impacts.

### School socioeconomic composition effects

Studies from a range of national contexts and methodological approaches have shown that school socioeconomic composition—i.e., the overall/average socioeconomic composition of students at a school—has a moderate to strong association with student outcomes, predicting student outcomes above and beyond that predicted by individual SES. Studies have shown that regardless of one’s individual SES, going to a school with a higher socioeconomic composition is related to higher academic achievement. These include studies conducted with large national datasets that examine the unique contribution of school SES for predicting student outcomes (Owens et al., 2016; Reardon et al., 2019; Willms, 1986), notably a meta-regression of 30 studies from OECD countries by van Ewijk and Sleegers (2010), and Sirin’s (2005) meta-regression of more than 100 studies from the US. In Australia, school socioeconomic composition effects on student academic outcomes have been demonstrated by Chesters (2019), Chesters and Daly (2015, 2017), and Lamb and Fullarton (2002).

Increases in school socioeconomic composition (i.e., school SES) are positively related to outcomes for all students, regardless of their individual socioeconomic status (Organisation for Economic Cooperation and Development [OECD] 2016). In some studies, school SES is as strong a predictor of student outcomes as student SES (e.g., Rumberger & Palardy, 2005), while other studies have found that school SES is an even stronger predictor of student outcomes than student SES (Borman & Dowling, 2010; Opdenakker & Van Damme, 2007; Owens et al., 2016; Sirin, 2005).

While most school socioeconomic composition studies are cross-sectional and therefore do not provide a strong basis for causal inference, a few longitudinal studies have been conducted. Palardy’s longitudinal study (2013) established a causal relationship between school socioeconomic composition and US student outcomes. Halpern-Manners (2016) found that students who were continuously exposed to high poverty schools from kindergarten to eighth grade had lower reading and mathematics scores relative to students who were continuously exposed to low poverty schools. Schwartz (2010) found that high poverty students who were randomly assigned to low poverty schools enjoyed substantial gains in reading and math over the course of seven years of primary school, and that their gains were substantially larger than high poverty students who were assigned to schools with greater numbers of high poverty students. On the other hand, some studies have shown small or no effects of school SES on student outcomes. These include Marks (2015), who found very small effects of school SES on numeracy and literacy skills in Australia; Wodkte and Parbst (2017), who found that school poverty was unrelated to literacy and problem-solving test scores for students from childhood through adolescence; and Lauen and Gaddis (2013), who found that classroom poverty was not associated with test scores. It is plausible that various longitudinal studies have found different conclusions because they have used different analytical approaches, measures of school socioeconomic composition, as well as different contexts. More longitudinal research is needed to establish causal effects but debates about appropriate approaches are ongoing (Sciffer et al., 2020; Thrupp, 1995).

The mechanisms by which school SES impacts achievement are multiple and complex. School SES is related with several factors that are associated with student outcomes. These factors include, for example, school material and human resources (Chiu & Khoo, 2005); teacher experience, effectiveness and qualifications (Chiu & Khoo, 2005; Darling-Hammond, 2010); classroom disciplinary climate and learning environments (Willms, 1999, 2010); peer effects (Harris, 2010; Palardy, 2013); and curriculum and instruction (Anyon, 1981; Willms, 2010). Higher SES schools often have learning environments that are better able to promote student outcomes, teaching, and learning compared to schools with lower SES compositions.

The literature suggests that peer ability effects may be stronger for low-achieving students than for their higher-achieving peers. In Sweden, Sund (2009) found that an increase in peer achievement benefits all students in the classroom but that the relation is not linear, with lower-achieving students benefiting more than their higher-achieving peers. Similar results were found in the US by Hanushek et al. (2001), who found that school achievement effects are stronger for low-performing students than for their higher-performing peers. Related to these findings is the impact of low-achieving students on their peers. In Israel, Lavy et al. (2011) found that the proportion of low-achieving students has a negative effect on the performance of other students. While these studies suggest that attending a school with a high overall level of achievement benefits lower-achieving students compared to their higher achieving peers, they do not provide evidence about our primary research question, namely whether school socioeconomic composition effects vary in strength depending on the performance level of the student.

### Theoretical perspectives

High-performing students tend to come from higher SES backgrounds, and high-performing schools tend to overwhelming enrol mostly students from high SES backgrounds (Gorard, 2006; Marsh, 1991; Marsh & Parker, 1984). In Australia, academically selective high schools enrol almost exclusively students from the highest socioeconomic backgrounds (Rowe & Perry, 2022). Academic performance and socioeconomic status are correlated positively because students from higher socioeconomic backgrounds experience more pro-schooling cultural, social, human, and financial capital at home. Moreover, these advantages for students from high SES backgrounds, and disadvantages for students from low SES backgrounds, compound and accumulate over time. In the case of learning, initial small differences grow larger over time because progression from each step to the next depends on attainment of satisfactory performance in the previous step and fosters further relative advantages or obstacles (DiPrete & Eirich, 2006; Merton, 1968).

The effect of school socioeconomic composition on the academic achievement of students from varying performance levels is not clear. It is possible that higher-performing students are less sensitive to school socioeconomic composition effects, and school practices more generally, because they enjoy many supports and resources from home, as well as from their individual capacities, that buffer them from practices at school. On the other hand, a completely alternative dynamic could be at play. Because of their high levels of capacity and motivation, high-performing students may be more likely to benefit from school practices than their lower-performing peers. According to this “it takes money to make money” line of thinking, high-performing students could be just as affected by school socioeconomic composition, or even more than their lower performing peers. Another possibility is that the effect is similar for all students, regardless of their performance level. Our study tests these possibilities with data on Australian youth.

### Australian context

Australia is a prosperous country with very high levels of economic and social development (United Nations Development Programme [UNDP] 2019). Schooling is primarily the responsibilities of the states and territories, but the federal government sets the national curriculum, administers standardised national testing, provides public reporting of school performance data, and is the main public funder of private schools. The main groups of students who face educational and social disadvantage are those from Indigenous backgrounds, youth from low socioeconomic backgrounds, and those who reside in rural/regional locations (Warren & Edwards, 2017). These three groups often overlap and disadvantages cumulate, with low SES Indigenous students from rural/remote communities typically exhibiting the highest levels of educational disadvantage and lowest performance.

Australian schooling is characterised by high levels of socioeconomic segregation. It has the fifth highest level of school socioeconomic segregation among member countries of the OECD, after Mexico, Chile, Hungary and the Czech Republic (OECD, 2019a). Mexico and Chile have high levels of income inequality and poverty, and Hungary and the Czech Republic have non-comprehensive, academically selective secondary schooling; both factors explain these four countries’ high levels of school social segregation. Australia, however, has low levels of poverty, low to moderate income inequality, and comprehensive (non-selective) secondary schooling for the vast majority of students.^1^ It has the highest level of school socioeconomic segregation among OECD countries that have comprehensive secondary schooling and low to moderate levels of income inequality.

High levels of school segregation in Australia are due, in large part, to its large private school sector. This relationship is consistent with a key factor in segregation in other nations (Alegre & Ferrer, 2010; Bonal & Bellei, 2018). Approximately 34% of all primary or secondary students attend a private (non-government) school, and this number increases to 41% among secondary students (Australian Bureau of Statistics, 2018). All non-government schools in Australia charge tuition fees as well as receive public funding.^2^ This means that Australia has one of the highest proportions of students among economically developed countries that attend a fee-charging school. On average, non-government schools enrol a larger proportion of socially advantaged students compared to government schools (Connors & McMorrow, 2015). Almost all low SES schools are public, and almost all high SES schools are private. Australia has the second highest degree of socioeconomic segregation between public and private schools among member countries of the OECD, second only to Spain (OECD, 2019a). Australian schooling also has high levels of school choice and competition, second only to Belgium in the proportion of students that attend a school that competes with at least one other local school (OECD, 2019a). Taken together, these dynamics suggest that Australia’s high level of school segregation is due, in part at least, to its high level of school choice and competition, which is fuelled by its large private school sector.

## Method

We used 2018 Australian data from the Programme for International Student Assessment (PISA) to conduct separate quantile and OLS regressions for the three subject domains (reading, mathematics, science) to answer our research questions.

1. Does the effect of school SES on PISA scores vary by student performance level?
2. What is the size of the school SES effect relative to the size of other school and student factors, including student SES, within different achievement quintiles?
3. Does the ratio of school SES effect size relative to family SES effect size vary by student performance quintiles?

Details about the dataset and analytical approach are provided below.

### Data

PISA is a large-scale international assessment administered to a two-stage stratified sample of schools and students in all OECD member countries as well as other participating countries. Students are aged from 15 years and 3 months to 16 years and 2 months of age at the beginning of the time of testing (OECD, 2018b). In Australia, most students who participate in PISA are in the 10th year of schooling. PISA has been administered every three years since 2000. We use data from the 2018 cycle, as it is the most recent (administration of the PISA 2021 cycle has been postponed to 2022 due to the Covid-19 pandemic). The aim of PISA is to assess young people’s reading, mathematical and scientific literacy, rather than assess their mastery of curricula or disciplinary knowledge. PISA evaluates students’ capacity to apply knowledge to solve problems and to understand everyday scenarios that are commonly encountered in modern societies.

The Australia PISA 2018 sample is nationally representative and includes 14,273 students sampled from a representative pool of 763 school buildings. Among these buildings, there were 21 where the size of the sample from the building was at least one standard deviation lower than the average building sample size. These potentially “under-sampled” 21 buildings offered an aggregate of 260 students to the overall data. Excluding these students and thus their buildings made nearly no difference in our empirical estimates. We therefore retained the full sample to maintain national representativeness as the building level.

**Fig. 1:**
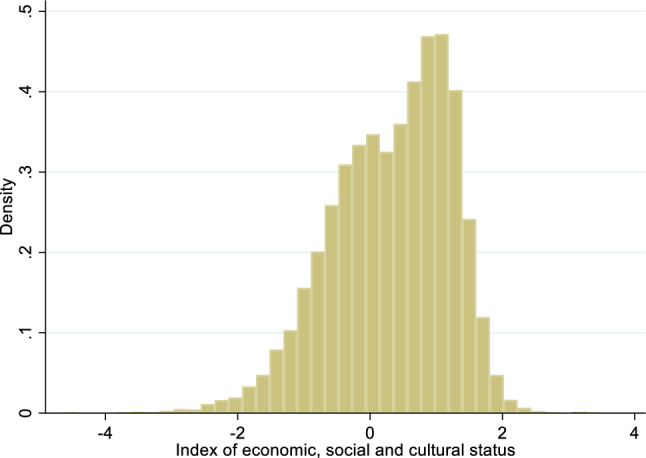
Distribution of student index of economic, social and cultural status (family SES indicator)

Fifty percent of students were female, 35% percent were born outside of Australia, and 13% percent spoke a language at home other than English. The operationalization of individual socioeconomic status is comprised of PISA’s index of economic, social and cultural status (ESCS); we refer to this variable as SES for comparability with the research literature and provide more detail in a later section. Higher values correspond to higher SES. The average SES was 0.32 (sd = 0.90). Figure 1 shows the distribution of ESCS. Student’s gender, native/immigrant status, language spoken at home, grade level, and SES are used as controls in multivariate models. The data contained information on 8th through 12th graders, but there were only nine 8th graders and five 12 graders. We excluded these 14 students from the analysis, reducing our effective sample size to 14,259, involving 9th, 10th, and 11th graders. In addition, while immigrant status and language spoken at home sometimes overlap, they are not the same variable. Some immigrants to Australia come from English-speaking backgrounds, and some native-born Australians speak a language at home other than English.

In addition to student controls, multiple school features are also used as covariates. These include school size, school sector (public or private), and school location. The average school building has a total enrolment of about 1,040 students with a standard deviation of 530.40. About 44 percent of students are enrolled in private schools. About five percent of students are enrolled in schools located in a village (population smaller than 3000), nine percent are in schools located in a small town (population 3000–15,000), 17 percent are in schools located in a medium to large town (15,000–100,000), 29 percent were in schools in a city (100,000–1,000,000), and nearly 40 percent were in schools in a large city (1,000,000+). Missing observations are a minor problem in PISA data, with negligible effects on our measures. We nonetheless imputed values for missing data using STATA 17’s MI (multiple imputation) procedure. Measures with complete data were used as predictors for imputation in a stepwise fashion. This procedure resulted in minor differences in our key findings.

**Fig. 2:**
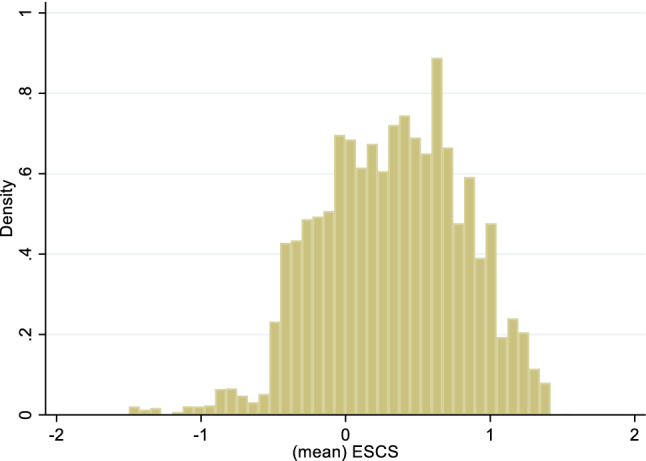
Distribution of mean school index of economic, social and cultural status (school SES indicator)

The central predictor of theoretical interest in this study is school SES. It is the building mean calculated from the individual family SES measure for all students in a given school who participated in PISA. School SES is typically measured by aggregating the SES of the students in a school or class (Willms, 2010), the approach that is widely used in studies that examine school socioeconomic composition effects (for example, see Benito et al. (2014) and Sciffer et al. (2020).^3^ The weighted average school SES (mean student ESCS by building) was 0.32 (sd = 0.49).The distribution appears in Fig. 2.

We use mathematics, reading, and science scores on PISA 2018 as our outcome measures. PISA uses item-response theory models to create standardized measures, scaled to fit approximately normal distributions. Mean scores are around 500 points, with standard deviations of 100 points (OECD, 2019b).

### Analytical approach

To investigate our motivating questions, we fitted multiple quantile regression (QREG) models to examine whether the relationship of school SES to student achievement varied across different quintiles of the achievement distribution. Quantile regression is useful for detecting whether predictors vary in strength for different quantiles on the dependent variable (Koenker & Hallock, 2001). In each of the three achievement domains (mathematics, reading, and science), ten plausible values of achievement were used in estimation. Any estimation of effects on a given achievement measure draws on all ten plausible values, utilizing the relevant trimmed and non-response adjusted student weight, a total of 80 replicate BRR (“balanced repeated replication”) weights, and 0.5 Fay adjustment (Jerrim, 2014; Jerrim et al., 2017; OECD, 2009). Within each quintile we estimated models that included six student controls (gender, language at home, immigrant status, Indigeneity, grade level, family SES) and four school controls (location type, sector, size, and SES). We also included a cross-level interaction term composed of school SES and student SES in order to estimate, within each quintile, how the school-level SES effect changed based on student SES. Finally, given the nesting of students within schools, we report standard errors for all coefficients clustered at the school level.

The quintile regression procedure involved multiple steps:

We regress each of the 10 plausible values for achievement on the predictors.

For each predictor’s estimated coefficient, we pool all 10 estimates (i.e., produce the average of all 10 effect estimates) to generate a *pooled_beta*.

For each predictor’s estimated coefficient, we pool all 10 standard error estimates (i.e., produce the average of all 10 standard errors) to create a pooled standard error (*pooled_stderr).*

We determine the measurement variance (*me*), which is the sum of the squared differences for all 10 individual coefficients from the pooled*_beta*, divided by nine (total number of plausible values minus one).

We calculate the sampling variance (*sv*) for the *pooled_beta*. It is the squared *pooled_stderr*.

We determine total variance (*tv*) for *pooled_beta* by combining *me* and *sv*. This is accomplished by *pooled_stderr*^*2*^ + ((1 + (1/10))*me).

We calculate the final standard error (*se*) for *pooled_beta* by taking the square root of total variance: *tv*^*(1/2)*^.

For each QREG model, we repeated these seven steps four times for each achievement domain because we estimated effects for 20th, 40th, 60th, and 80th quintiles. This resulted in four coefficient (*pooled_beta*) and standard error (*se*) pairs for each predictor involved, in each achievement domain.

We also conducted ordinary least squares regression (OLS) analyses for students within each achievement quintile using the same dependent and independent variables. We estimated the OLS models only once per achievement domain (quintile). We then statistically compared findings from the quantile regression models to those from the OLS models. Unlike quantile regression, OLS assumes uniform effects across different quintiles of the achievement distribution. This helps statistically test the utility of quantile regression findings over findings from OLS.

We report estimates based on both unstandardized and standardized data. The former helps interpret effects in terms of the raw scales of our measures. The latter facilitates size comparisons across coefficients. Since we standardized the data for the entire country, our measures are by nature grand mean-centred.

## Results

To answer the paper’s motivating research questions, we conducted a quantile regression of student achievement in mathematics, reading, and science arrayed from highest to lowest quintiles, as described in the previous section. We also conducted an OLS regression using the same set of predictors. OLS regression, by definition, is insensitive to quantile-specific differences in the predictors’ effects (i.e., it assumes the same effect for a given predictor across all quintiles). Thus, comparing OLS estimates to quantile regression estimates helps determine the potential value that quantile regression brings to the analysis and to the related inferences. The results of our OLS and quantile regression analyses of PISA mathematics, reading, and science achievement appear in Tables 1, 2, 3. Each table presents the OLS findings (Models 1 and 6) and quantile regression results by the 20th, 40th, 60th, and 80th quintiles (Models 2–5 and Models 7–10). Models 2 through 5 present results as unstandardized coefficients while Models 7 through 10 present the standardized coefficients.

The findings indicate that various student level control variables have significant effects on mathematics, reading, and science PISA scores. Consistent with other national contexts, being male is positively associated with mathematics and science but negatively with reading (Mullis et al., 2017; OECD, 2016). Being an immigrant has a uniformly positive effect on mathematics, reading, and science performance, a finding that is consistent with other analyses of PISA (OECD, 2018a; Thomson et al., 2019)—though the effect is marginal in size for science. This “immigrant advantage” is likely due to two interrelated factors. First, Australia’s skills-based immigration policy prioritises immigrants with high levels of educational attainment and occupational status. Second, many immigrants, especially those who voluntarily migrate to multicultural immigrant countries such as New Zealand, the US, Canada and Australia, possess attributes and aspirations that promote school success (Abramitzky & Boustan, 2022; Ogbu, 1998; Portes & Rimbaut, 2014). Being an Indigenous Australian and not speaking English in the home have significant negative relationships with performance in all three subjects. The “Indigenous status” effect is uniquely revealing in the Australian context, an issue to which we return below. Unsurprisingly, the higher the grade level, the more successful the student on the PISA test given the greater accumulated learning in all three subjects across the grade span. As expected, family SES is positively related to test scores regardless of substantive area.

At the school level, school size and school SES have positive relationships with performance in all subject areas. Notably, school location and school sector have no significant effect on PISA scores. Importantly, the interaction of school SES with student SES also has a strong positive effect on performance in all three areas. As we shall address below, this indicates that the school SES effect on the average student’s achievement is greater when the student’s SES is higher.

The OLS and unstandardized quantile regression coefficients permit us to answer the first research question concerning whether school SES predicts school performance in mathematics, reading, and science for Australian students at different levels of performance, net of controls for other individual-level and school-level factors. The standardized quantile regression results permit us to answer the second research question concerning the relative influence of school SES across the various performance quintiles. Standardized coefficients indicate whether the relative influence of school SES varies by performance quintile in its relationship to the other significant factors that predict achievement. Put another way, when we compare the size of the standardized coefficients within a given quintile, do we find that the *relative* influence of school SES on performance varies by performance quintile?

Our results indicate that achievement levels in mathematics, reading, and science are associated with mean school SES along with school size, and students’ gender, nativity, home language, grade level, and family SES. Notably, irrespective of performance quintile, school SES has a significant positive effect on PISA scores net of student and other school control variables. The range of the unstandardized coefficients across the quintiles was modest for all three PISA tests. For example, in Table 1, Models 2 through 5 indicate that the unstandardized coefficients for the relationship of school ESCS to mathematics PISA score range from 43.937*** to 45.918***. Models 2 through 5 in Table 2 indicate that the unstandardized coefficient for the relationship of school ESCS to reading PISA score range 39.898*** to 47.828***, and Models 2 through 5 in Table 3 indicate that the unstandardized coefficients for the relationship of school ESCS to science PISA scores range from 42.244*** to 46.383***. Additionally, the ESCS regression coefficients in each quintile are within the confidence intervals for the complementary OLS regression (Model 1 in Table 1 for mathematics: 46.542***; Model 1 in Table 2 for reading 43.131***; Model 1 in Table 3 for science 44.444***). Together, these OLS results indicate that the effect of school SES does not vary by student performance level. ***The association between achievement score and school SES is relatively the same or similar for all students, regardless of their performance level.*** The school SES effect is effectively the same for high-performing students as it is for lower-performing students. These results suggest that school SES’s role in test performance is relatively as important for the highest performing students (80%ile) as for the lowest performing pupils (20%ile). Thus, based on the OLS results we conclude that irrespective of their performance quintile, all Australian students’ mathematics, reading, and science achievement scores are significantly and similarly related to the mean SES of the school they attend.

**Table 1:** Full model math

	Unstandardized					Standardized				
	OLS	Quantile regression				OLS	Quantile regression			
	Model 1	Model 2	Model 3	Model 4	Model 5	Model 6	Model 7	Model 8	Model 9	Model 10
		20th quintile	40th quintile	60th quintile	80th quintile		20th quintile	40th quintile	60th quintile	80th quintile
Student										
Male	9.786***	6.090*	9.438***	12.188***	13.435***	0.106***	0.066*	0.102***	0.132***	0.146***
	(2.831)	(3.649)	(3.561)	(3.816)	(3.461)	(0.030)	(0.039)	(0.038)	(0.041)	(0.038)
Not born in Australia	15.663***	12.073***	15.947***	18.380***	17.539***	0.170***	0.131***	0.173***	0.200***	0.191***
	(2.672)	(4.499)	(4.129)	(3.850)	(3.490)	(0.029)	(0.048)	(0.044)	(0.042)	(0.038)
Indigenous	− 38.538***	− 40.009***	− 37.923***	− 36.851***	− 36.812***	− 0.419***	− 0.435***	− 0.412***	− 0.401***	− 0.400***
	(6.005)	(8.629)	(9.476)	(7.625)	(7.749)	(0.064)	(0.092)	(0.101)	(0.082)	(0.084)
Non-English at home	− 13.432***	− 18.847***	− 18.025***	− 12.695**	− 5.560	− 0.146***	− 0.205***	− 0.196***	− 0.138**	− 0.060
	(4.444)	(6.472)	(5.379)	(5.773)	(6.498)	(0.048)	(0.070)	(0.058)	(0.062)	(0.070)
Grade 10 (v. 9)	23.201***	25.476***	23.800***	22.604***	22.443***	0.252***	0.277***	0.259***	0.246***	0.244***
	(4.272)	(6.354)	(4.974)	(4.878)	(5.337)	(0.045)	(0.068)	(0.053)	(0.052)	(0.057)
Grade 11 (v. 9)	52.109***	51.340***	52.427***	52.854***	53.010***	0.567***	0.558***	0.570***	0.575***	0.577***
	(6.587)	(10.200)	(8.796)	(7.352)	(8.090)	(0.070)	(0.109)	(0.095)	(0.079)	(0.088)
ESCS	16.219***	14.674***	17.411***	18.668***	18.681***	0.159***	0.144***	0.171***	0.184***	0.184***
	(1.461)	(2.150)	(2.176)	(2.314)	(1.953)	(0.014)	(0.021)	(0.021)	(0.022)	(0.019)
School										
Small town (v. village)	0.974	3.756	8.021	− 0.129	− 2.083	0.010	0.041	0.087	− 0.001	− 0.022
	(8.604)	(12.497)	(12.242)	(10.754)	(11.358)	(0.093)	(0.136)	(0.133)	(0.117)	(0.123)
Town (v. village)	− 0.407	0.956	3.508	− 1.960	− 3.570	− 0.004	0.010	0.038	− 0.021	− 0.038
	(8.478)	(11.257)	(11.880)	(10.264)	(11.595)	(0.092)	(0.122)	(0.129)	(0.111)	(0.126)
City (v. village)	− 4.196	− 0.982	0.954	− 5.218	− 8.780	− 0.045	− 0.010	0.010	− 0.056	− 0.095
	(8.951)	(11.940)	(11.818)	(10.863)	(12.064)	(0.097)	(0.130)	(0.128)	(0.118)	(0.131)
Large city (v. village)	− 3.936	− 2.795	0.044	− 5.483	− 6.722	− 0.042	− 0.030	0.000	− 0.059	− 0.073
	(8.346)	(11.023)	(11.610)	(10.340)	(10.373)	(0.090)	(0.120)	(0.126)	(0.112)	(0.113)
Private school	− 4.933	0.803	− 1.370	− 3.815	− 8.571	− 0.053	0.008	− 0.014	− 0.041	− 0.093
	(4.961)	(5.888)	(5.428)	(5.691)	(5.792)	(0.053)	(0.064)	(0.059)	(0.061)	(0.063)
School size	0.007**	0.010**	0.009***	0.007**	0.005	0.045**	0.060**	0.054***	0.045**	0.033
	(0.003)	(0.004)	(0.003)	(0.003)	(0.004)	(0.019)	(0.027)	(0.021)	(0.022)	(0.025)
School ESCS	46.542***	43.937***	43.299***	44.284***	45.918***	0.250***	0.236***	0.232***	0.238***	0.246***
	(5.700)	(6.341)	(6.205)	(6.464)	(6.281)	(0.030)	(0.034)	(0.033)	(0.035)	(0.034)
School ESCS*Student ESCS	12.468***	15.231***	13.651***	12.045***	11.208***	0.076***	0.093***	0.083***	0.073***	0.068***
	(2.585)	(3.684)	(3.595)	(3.942)	(4.030)	(0.015)	(0.022)	(0.022)	(0.024)	(0.024)
Constant	435.551***	360.845***	408.391***	457.175***	509.520***	− 0.246***	− 1.034***	− 0.528***	− 0.013	0.537***
	(9.082)	(13.040)	(12.539)	(10.241)	(12.021)	(0.097)	(0.143)	(0.134)	(0.113)	(0.132)
R^2^	0.205								0.205	
Pseudo-R^2^		0.106	0.113	0.114	0.115		0.106	0.113	0.114	0.115

**Table 2:** Full model reading

	Unstandardized					Standardized				
	OLS	Quantile regression				OLS	Quantile regression			
	Model 1	Model 2	Model 3	Model 4	Model 5	Model 6	Model 7	Model 8	Model 9	Model 10
		20th quintile	40th quintile	60th quintile	80th quintile		20th quintile	40th quintile	60th quintile	80th quintile
Student										
Male	− 27.635***	− 34.329***	− 28.075***	− 24.523***	− 19.545***	− 0.254***	− 0.315***	− 0.258***	− 0.225***	− 0.179***
	(2.406)	(4.495)	(3.676)	(3.239)	(3.752)	(0.022)	(0.041)	(0.033)	(0.029)	(0.034)
Not born in Australia	10.283***	10.966***	13.848***	11.318***	10.210***	0.094***	0.100***	0.127***	0.104***	0.093***
	(2.915)	(4.667)	(4.003)	(3.846)	(3.940)	(0.026)	(0.042)	(0.036)	(0.035)	(0.036)
Indigenous	− 44.624***	− 45.579***	− 47.914***	− 47.473***	− 40.376***	− 0.410***	− 0.419***	− 0.440***	− 0.436***	− 0.371***
	(5.702)	(10.690)	(7.717)	(8.889)	(9.038)	(0.052)	(0.098)	(0.070)	(0.082)	(0.083)
Non-English at home	− 26.798***	− 36.247***	− 30.993***	− 23.264***	− 20.063***	− 0.246***	− 0.333***	− 0.284***	− 0.213***	− 0.184***
	(4.201)	(6.135)	(5.924)	(5.290)	(6.253)	(0.038)	(0.056)	(0.054)	(0.048)	(0.057)
Grade 10 (v. 9)	26.795***	29.586***	28.805***	25.495***	26.560***	0.246***	0.272***	0.264***	0.234***	0.244***
	(3.746)	(5.821)	(5.407)	(5.826)	(5.735)	(0.034)	(0.053)	(0.049)	(0.053)	(0.052)
Grade 11 (v. 9)	57.419***	60.504***	60.228***	57.477***	56.267***	0.527***	0.556***	0.553***	0.528***	0.517***
	(5.151)	(7.764)	(7.073)	(8.619)	(7.506)	(0.047)	(0.071)	(0.064)	(0.079)	(0.068)
ESCS	19.466***	17.332***	21.452***	23.132***	21.751***	0.162***	0.144***	0.178***	0.192***	0.181***
	(1.526)	(2.808)	(2.031)	(2.285)	(2.245)	(0.012)	(0.023)	(0.016)	(0.019)	(0.018)
School										
Small town (v. village)	− 2.563	− 4.246	3.497	4.755	− 9.060	− 0.023	− 0.039	0.032	0.043	− 0.083
	(7.859)	(12.008)	(9.725)	(11.233)	(14.266)	(0.072)	(0.110)	(0.089)	(0.103)	(0.131)
Town (v. village)	3.509	5.137	9.324	3.329	− 3.732	0.032	0.047	0.085	0.030	− 0.034
	(7.259)	(10.782)	(9.047)	(10.730)	(13.206)	(0.066)	(0.099)	(0.083)	(0.098)	(0.121)
City (v. village)	− 0.713	− 0.310	5.139	2.865	− 9.794	− 0.006	− 0.002	0.047	0.026	− 0.090
	(7.596)	(10.526)	(8.752)	(11.125)	(13.572)	(0.069)	(0.096)	(0.080)	(0.102)	(0.124)
Large city (v. village)	− 0.438	− 1.283	3.553	2.356	− 6.324	− 0.004	− 0.011	0.032	0.021	− 0.058
	(7.091)	(10.009)	(8.574)	(10.952)	(13.486)	(0.065)	(0.092)	(0.078)	(0.100)	(0.124)
Private school	− 3.141	− 0.362	− 1.752	− 4.225	− 7.415	− 0.028	− 0.003	− 0.016	− 0.038	− 0.068
	(4.551)	(6.427)	(5.278)	(5.458)	(5.448)	(0.041)	(0.059)	(0.048)	(0.050)	(0.050)
School size	0.009***	0.010***	0.008**	0.009***	0.006*	0.044***	0.053***	0.042**	0.045***	0.032*
	(0.002)	(0.004)	(0.003)	(0.003)	(0.003)	(0.014)	(0.020)	(0.018)	(0.018)	(0.017)
School ESCS	43.131***	47.828***	45.928***	39.898***	40.235***	0.195***	0.217***	0.208***	0.181***	0.182***
	(5.326)	(7.122)	(6.095)	(6.551)	(7.164)	(0.024)	(0.032)	(0.027)	(0.029)	(0.032)
School ESCS*Student ESCS	12.752***	15.077***	12.674***	12.063***	9.861***	0.065***	0.077***	0.065***	0.062***	0.050***
	(2.353)	(3.701)	(3.501)	(3.667)	(3.845)	(0.012)	(0.019)	(0.017)	(0.018)	(0.019)
Constant	462.385***	376.312***	430.379***	486.155***	554.683***	− 0.060	− 0.820***	− 0.345***	0.158	0.753***
	(7.942)	(10.640)	(9.490)	(11.854)	(14.610)	(0.072)	(0.100)	(0.088)	(0.113)	(0.133)
R^2^	0.191					0.191				
Pseudo-R^2^		0.116	0.112	0.103	0.091		0.116	0.112	0.103	0.091

**Table 3:** Full model science

	Unstandardized					Standardized				
	OLS	Quantile regression				OLS	Quantile regression			
	Model 1	Model 2	Model 3	Model 4	Model 5	Model 6	Model 7	Model 8	Model 9	Model 10
		20th quintile	40th quintile	60th quintile	80th quintile		20th quintile	40th quintile	60th quintile	80th quintile
Student										
Male	6.648***	0.891	5.927	9.911***	13.055***	0.066***	0.008	0.058	0.098***	0.130***
	(2.584)	(4.117)	(3.623)	(2.979)	(3.341)	(0.025)	(0.040)	(0.035)	(0.029)	(0.033)
Not born in Australia	5.054*	4.400	5.134	5.673	5.621	0.050*	0.043	0.051	0.056	0.055
	(2.943)	(4.386)	(3.887)	(4.060)	(4.942)	(0.029)	(0.043)	(0.038)	(0.040)	(0.049)
Indigenous	− 46.124***	− 48.864***	− 49.103***	− 47.195***	− 42.735***	− 0.459***	− 0.486***	− 0.488***	− 0.469***	− 0.425***
	(5.795)	(8.507)	(9.782)	(9.061)	(8.358)	(0.057)	(0.083)	(0.096)	(0.089)	(0.083)
Non-English at home	− 20.794***	− 27.580***	− 24.788***	− 20.950***	− 16.531***	− 0.207***	− 0.274***	− 0.246***	− 0.208***	− 0.164***
	(4.701)	(6.131)	(6.777)	(5.375)	(6.921)	(0.046)	(0.061)	(0.067)	(0.053)	(0.068)
Grade 10 (v. 9)	25.470***	28.952***	24.344***	24.775***	25.756***	0.253***	0.288***	0.242***	0.246***	0.256***
	(3.753)	(5.603)	(4.998)	(5.373)	(5.973)	(0.037)	(0.055)	(0.049)	(0.053)	(0.059)
Grade 11 (v. 9)	50.759***	53.090***	50.208***	52.079***	53.731***	0.505***	0.528***	0.499***	0.518***	0.534***
	(5.570)	(9.461)	(8.379)	(7.143)	(9.449)	(0.054)	(0.093)	(0.081)	(0.070)	(0.093)
ESCS	17.762***	15.609***	19.844***	21.218***	19.802***	0.160***	0.140***	0.178***	0.191***	0.178***
	(1.421)	(2.694)	(2.466)	(2.210)	(2.199)	(0.012)	(0.024)	(0.022)	(0.019)	(0.019)
School										
Small town (v. village)	− 0.506	3.644	5.716	− 2.549	− 5.684	− 0.005	0.036	0.056	− 0.025	− 0.056
	(8.573)	(11.334)	(11.253)	(11.865)	(12.363)	(0.085)	(0.112)	(0.111)	(0.118)	(0.122)
Town (v. village)	1.435	5.933	9.084	− 1.094	− 7.552	0.014	0.058	0.090	− 0.010	− 0.075
	(8.359)	(11.471)	(10.765)	(11.754)	(13.638)	(0.083)	(0.114)	(0.107)	(0.117)	(0.135)
City (v. village)	− 1.654	1.087	5.501	− 0.757	− 9.443	− 0.016	0.010	0.054	− 0.007	− 0.094
	(8.711)	(12.039)	(11.277)	(12.176)	(13.207)	(0.086)	(0.119)	(0.112)	(0.121)	(0.131)
Large city (v. village)	− 3.145	− 3.514	2.945	− 3.185	− 7.601	− 0.031	− 0.035	0.029	− 0.031	− 0.075
	(8.179)	(11.138)	(10.801)	(11.539)	(12.685)	(0.081)	(0.111)	(0.107)	(0.114)	(0.126)
Private school	− 5.520	− 1.337	− 3.502	− 4.706	− 8.530	− 0.054	− 0.013	− 0.034	− 0.046	− 0.084
	(4.953)	(6.248)	(5.848)	(6.057)	(6.226)	(0.049)	(0.062)	(0.058)	(0.060)	(0.061)
School size	0.006**	0.010***	0.008***	0.005	0.002	0.036**	0.056***	0.047***	0.028	0.014
	(0.003)	(0.004)	(0.003)	(0.003)	(0.004)	(0.016)	(0.022)	(0.019)	(0.020)	(0.022)
School ESCS	44.444***	46.383***	44.784***	42.591***	42.244***	0.218***	0.228***	0.220***	0.209***	0.207***
	(5.603)	(7.644)	(6.207)	(7.121)	(7.266)	(0.027)	(0.037)	(0.030)	(0.034)	(0.035)
School ESCS*Student ESCS	11.556***	17.635***	13.713***	9.343***	7.161*	0.064***	0.098***	0.076***	0.052***	0.039*
	(2.450)	(3.995)	(3.288)	(3.640)	(3.716)	(0.013)	(0.022)	(0.018)	(0.020)	(0.020)
Constant	453.294***	365.080***	421.862***	479.786***	542.065***	− 0.188**	− 1.006***	− 0.465***	0.057	0.636***
	(8.785)	(11.982)	(11.815)	(12.408)	(12.672)	(0.086)	(0.119)	(0.119)	(0.130)	(0.128)
R^2^	0.170					0.170				
Pseudo-R^2^		0.099	0.102	0.095	0.084		0.099	0.102	0.095	0.084

As noted earlier, the strong and positive interaction effect of school SES with student SES indicates that the school-level SES effect is greater when the student’s SES is higher. For instance, as seen in Model 1 in Table 1, each unit of student SES increases the school-level SES effect by 12.468*** units. This *augmentation* pattern is observed in all three subject areas, meaning school SES has a more positive influence on achievement for students whose family SES is higher. On the flipside, less advantaged students benefit less from school SES composition. This reduced school SES effect may be due to how low student SES can limit the compositional advantages of school SES—for instance, adverse nonschool conditions for low-SES students can counteract beneficial school-level SES effects (Conley & Albright, 2004; Duncan & Murnane, 2011). It is also possible that school segregation by SES concentrates low-SES students into schools apart from higher SES peers, resulting in lower school-level SES effects for low-SES students. Both dynamics may be at play in varying degrees in the Australian context, an issue our data is unable to help tease out. Ultimately, our findings suggest that low-SES students are likely to benefit less from increased school SES, a cross-level dynamic that has received limited attention in past research. Finally, while the strong and positive school/student SES interaction is prevalent across all achievement quintiles (see Tables 2 and 3), it is somewhat reduced in size for high achievers, as shown by the pattern of interaction effects from Model 2 to Model 5 in any of the three tables. While these effects are within one another’s 95% confidence intervals in each table, there is a clear trend for the interaction effect to become smaller in higher achieving quintiles. Plausibly, the augmentation of the school-level SES effect by student SES is a somewhat smaller issue when the student is already higher achieving. Notably, as seen in Model 5 in Table 3, for science achievement, the school/student SES interaction effect is only borderline-significant in the 80^th^ quintile (7.161*).

To answer the second research question concerning the *relative* influence of school SES within performance quintiles, we examine the standardized coefficients within a quintile in Models 7 through 10 in each table. The first finding of interest is that the single largest factor predicting PISA scores for all three subjects across all quintiles is being in 11th grade. This is unsurprising given that 11th graders are likely to be academically more advanced than students in other lower grades in our data (9th and 10th graders), and that the PISA test is not necessarily grade-aligned. The grade level effect is followed by that of Indigenous status, which has a significant large negative relationship to scores.^4^ This finding is not surprising either, as Indigenous students in Australia have consistently suffered from high levels of unequal educational opportunities and outcomes. In the PISA 2018 cycle, the mean score of Indigenous students was substantially lower than their non-Indigenous peers, with the achievement gap roughly equivalent to two-and-a third years of schooling (Thomson et al., 2019). This very sizeable achievement has been documented over all PISA cycles (Thomson et al., 2019), and similarly sized achievement gaps have been uncovered in national data sets administered to primary and secondary students (Ford, 2012; Lamb et al., 2015). These sobering educational inequalities are the result of generations of disenfranchisement (De Plevitz, 2007), similar to that faced by First Nations peoples in other settler colonial contexts such as the US, Canada and New Zealand.

### Mathematics

Our presentation of findings begins with PISA mathematics scores for the lowest quintile and continues through the highest quintile (Models 7–10 in Table 1). We start with addressing school features, school SES in particular, which is the focus of our research question. We find that only school size effect and the main school-level SES effect are notable. The former effect is small (varying between 0.033 and 0.060** across quintiles, and is non-significant in the 80th quintile), but the latter is large and consistently greater than the student-level SES effect (varying between 0.232*** and 0.246***). The greater school SES effect relative to student SES effect is consistent with past literature. Also, as noted earlier, in reference to unstandardized estimates, the school SES effect varies little across quintiles and is statistically the same as the OLS baseline estimate.

The standardized estimates facilitate interpreting the size of the school SES main effect in combination with the school/student SES interaction effect. For instance, in Model 7, the main school SES coefficient (0.236**) represents the school SES effect on the student with average SES in Australia (i.e., when the standardized student SES equals to zero). Here the interaction effect (0.093**) indicates that the school SES effect grows by 0.093 standard deviation units for students whose SES is one standard deviation above the Australian mean. As noted earlier, in reference to unstandardized estimates, the interaction effect is somewhat smaller for higher achieving quintiles, though the differences remain statistically small.

Regarding student traits, we find that, net of the grade level effects (e.g., being in 11th grade has the largest effect), the size of the Indigenous status effect surpasses that of the effects of all other student background controls. The indigeneity effect is followed by the effects of not speaking English at home (varying between − 0.060 and − 0.205*** across quintiles), immigrant status (varying between 0.131*** and 0.200***), and SES (varying between 0.144*** and 0.184***). These effects are largely similar across quintiles and are also within the confidence interval of the corresponding OLS baseline estimate in Model 6. An exception is the effect of not speaking English at home, which gradually declines in consecutive models, losing significance in Model 10. This last estimate, 0.060 for the 80th quintile, is statistically different from estimates for 20th and 40th quintiles and is only marginally within the confidence interval of the OLS baseline effect (− 0.146***). Thus, the negative effect of not speaking English at home is weakest for high achievers compared to low achievers. We hypothesize that this somewhat counterintuitive finding may be mediated by the positive effect of socioeconomic status on achievement. High achieving students who do not speak English at home are likely to have parents who are highly educated and fluent in English, thereby neutralising the negative effect of not speaking English at home. Finally, the effect of being male is rather modest (varying between 0.066* and 0.146***). Notably, the male effect grows larger for higher achievers. For the 80^th^ quintile, it is significantly different than the effect for 20^th^ quintiles as well as the OLS baseline. Similar to our hypothesized explanation above, it is likely that the effect of being male on achievement is mediated by the intersection of gender and SES dynamics (Prieto-Rodriguez et al., 2020; Saw et al., 2018).

### Reading

We report the relative importance of student- and school-level factors for PISA reading scores by quintile beginning with the lowest quintile results (Models 7–10 in Table 2). Starting with school-level factors, we find, as before, that only the school size effect and the main school SES effect are notable. The former effect is small, but the latter is large and consistently greater than the student-level SES effect across all quintiles, varying between 0.181*** and 0.217***. As in the case of mathematics achievement, the school SES effect on reading is greater for higher SES students. Seen in Model 7 in Table 2, the school SES effect for the average SES student in 0.217***, which increases by 0.077*** (the interaction effect) to 0.294 for students whose SES is one standard deviation above the Australian mean. A similar pattern is observed for subsequent achievement quintiles, though the interaction effect tends to reduce in size for higher achieving groups.

Turning to student traits, we find that the large sizes of the effects of grade level and Indigenous status are followed by the sizes for effects of being male (varying between − 0.179*** and − 0.315*** across quintiles), not speaking English at home (varying between − 0.184*** and − 0.333***), family SES (varying between 0.144*** and 0.192***), and immigrant status (varying between − 0.093*** and − 0.127***). Notably, compared to that for mathematics, the male effect on reading is particularly strong, especially for low achievers. It is also larger than the baseline OLS estimate in Model 6.

### Science

As before, we report the relative importance of student- and school-level factors for PISA reading scores by quintile beginning with the lowest quintile results (Models 7–10 in Table 3). Our school-level estimates are similar to those for mathematics and reading. Only the school size effect and the main school SES effect are notable. The former effect is small, but the latter is large and consistently greater than the student-level SES effect across all models, varying in this case between 0.207*** and 0.228***. Once again, given the strong positive student/school SES interaction effect, the effect of school-level SES is greater for higher SES students. Similar to findings in previous tables, the interaction effect is somewhat smaller in higher achievement quintiles. Notably, however, unlike in the case of mathematics and reading, the interaction effect on science achievement is only borderline-significant in the 80^th^ quintile (0.039* in Model 10). This means that, for highest achievers in science, the school-level SES effect is largely uniform, meaning less contingent on student SES.

Regarding student traits, we find, as in previous tables, that the large sizes of grade level and Indigenous status effects are followed by the sizes for effects of not speaking English at home (varying between − 0.164*** and − 0.274*** across quintiles) and SES (varying between 0.140*** and 0.191***). Unlike for mathematics and reading, immigrant status does not have a large effect. Neither does gender, except for high achievers. For instance, the effect of being male in 80th quintile (0.130***) is significantly greater than in 20th and 40th quintiles, as well as relative to the baseline OLS estimate in Model 6.

### Summarizing the relative SES influence by quintile

While size and strength of the effects of certain student traits vary by substantive area as well as by quintile, the student- and school- level SES effects are relatively similar across all areas and quintiles. Invariably, the school SES effect is moderately larger than the associated student SES effect.

### Positing the school/home impact ratio

**Fig. 3:**
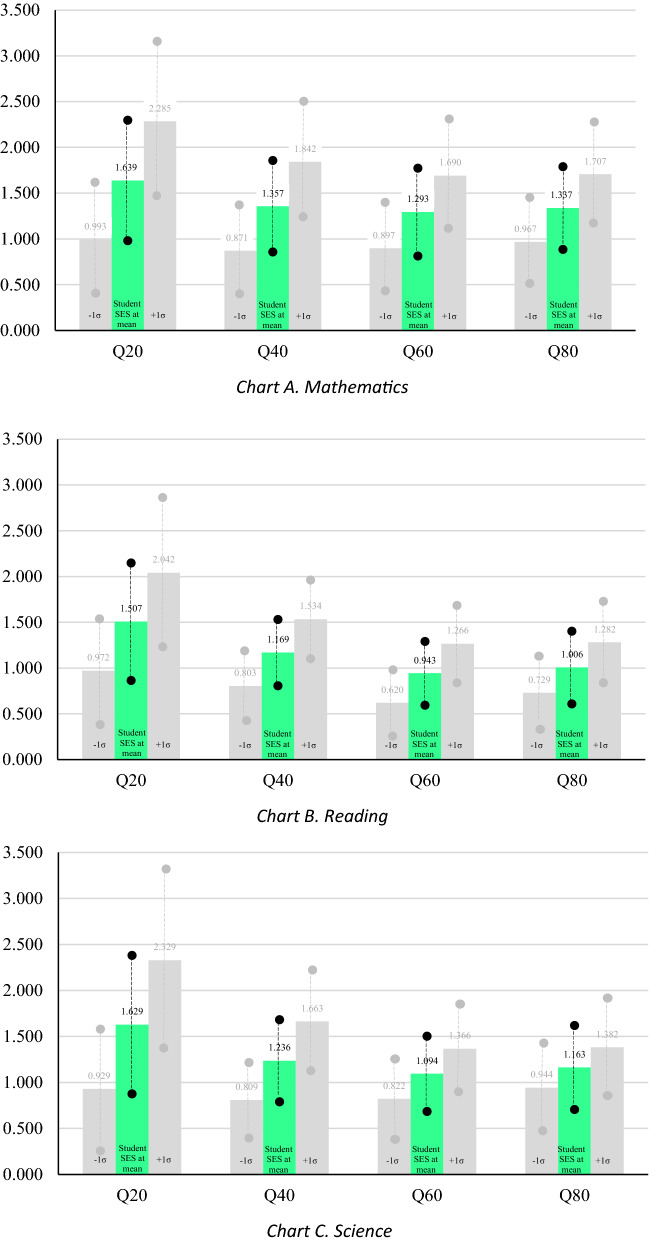
School/home impact ratios by achievement quintile

Much of the contemporary debate about achievement differences hinges on whether student or school factors contribute to outcomes. PISA data have both family and school SES measures. Given our interest in the relative effects of school SES on achievement, we created a ratio between the coefficient for school SES to family SES (school SES effect is the numerator and student SES effect is the denominator) within each quintile for mathematics, reading, and science PISA scores. Using the standardized coefficients in Tables 1, 2, 3, we calculate the ratio by taking the standardized school SES coefficient and dividing it by the family SES coefficient within each quintile. We refer to this ratio as the School/Home Impact Ratio (SHIR) and define it as *the impact of school SES on achievement relative to the impact of home SES within a quintile* controlling for other school and individual level factors. We present the SHIR for each quintile of mathematics, reading, and science performance in Fig. 3. Notably, given the school/student SES interaction in our multivariate models, we generate three SHIR estimates in a given quintile: (1) for zero student SES (students whose SES is at the Australian mean), (2) for − 1 student SES (students whose SES is one standard deviation below the Australian mean), and (3) for + 1 student SES (students whose SES is one standard deviation above the Australian mean). In Fig. 3, the dark shaded bars in the middle show the SHIR for the average SES student while the adjacent light shaded bars show SHIR for students whose SES is one standard deviation below/above the Australian average. Given the positive school/student SES interaction effect in all our tables, the height of any dark shaded bar is halfway between that of light shaded bars around it. For simplicity, we focus our discussion below on the dark shaded bars, for the average SES student.

We show, in Chart A for mathematics, that the school SES effect is 63.9% greater than the student SES effect in the 20th quintile (see 1.639 on the first dark shaded bar). This comes from Model 7 in Table 1, by dividing 0.236 (the main school SES effect) by 0.144 (student SES effect). We also calculated 95% confidence intervals for all SHIR estimates, based on standard errors for the numerator and denominator.^5^ These confidence intervals are shown by vertical lines on each bar. In all three academic areas that we address, SHIR is highest at the lowest achievement quintile, gradually decreasing in subsequent quintiles, though a modest recovery is observed at the 80th quintile. Broadly, this pattern suggests that the size of the school SES contribution to achievement, relative to the size of the student SES contribution, is largest for low achievers (note that the same pattern holds for light shaded bars). Our analysis provides modest statistical support for this finding as well, especially for reading and science. In Chart B (for reading), SHIR for the 20th quintile, 1.507, is outside the confidence interval of SHIR for the 60th quintile, 0.943. Though it is within the confidence interval of SHIR for the 40th quintile, it is near the top end of this interval. We observe the same exact pattern of contrasts in Chart C (for science). This pattern is important for future studies to further explore, including with data from other nations.

## Discussion

Our study uncovered four main findings. First, consistent with past work, we find that the school SES is a stronger predictor of academic achievement than is student SES. While this pattern observed for all three subject domains, it is particularly pronounced for mathematics, the most school-dependent domain among the three we address. As seen in Model 6 (OLS) in Table 1, the 95% confidence intervals for school and student SES effect estimates marginally overlap.^6^ The overlaps are greater in reading and science (see Model 6 in Tables 2 and 3), but the pattern is similar.

Second, the effect of school SES on academic achievement is the largely the same for all students, regardless of their level of academic performance. In other words, high-achieving students are just as sensitive to the effects of school SES as are their lower achieving peers. To the best of our knowledge, only one other study, Willms (1986), has examined whether the effects of school SES vary for students with different achievement levels. Conducted in Scotland, Willms (1986) also found that the school SES effect is similar for students from all achievement levels.

Third, we find that the school SES effect is larger for higher SES students regardless of achievement level in all three areas, though this pattern is somewhat less pronounced for high achievers. And fourth, our SHIR measure shows that the size of the school SES effect relative to the size of the student SES effect may be larger in lower performance quintiles. We observe this pattern descriptively, but also provide modest preliminary statistical support for it in the areas of reading and science.

Our findings provide two interrelated answers to the question asked in the title of our paper, “Does school SES matter less for high-performing students than for their lower-performing peers?” On the one hand, school SES is equally predictive of academic performance regardless of a student’s performance level; it does not have a differential effect for students of differing performance levels. In this sense, school SES “matters” the same for high-performing students as it does for their lower-performing peers. On the other hand, the relative importance of school SES is more impactful for lower-performing pupils compared to family SES. From this perspective, school SES “matters” more for lower-performing students. So, the answer to our question is both yes and no.

Cumulative (dis)advantage theory predicts that student outcomes will be affected by school socioeconomic composition along with other school and student characteristics such that any school-SES effects will be more harmful for marginalized youth and will increase the magnitude of the impact. Our findings lend support to these expectations. Specifically, we find that the school SES effect relative to student SES effect (what we refer to as the “school/home impact ratio” [SHIR]) is greater in lower achieving quintiles. This is precisely the dynamic predicted by cumulative (dis)advantage: the disadvantages of learning in socioeconomically segregated schools appear to cumulate given the other student and school factors that shape performance. Compounding the structural disadvantages associated with attending segregated low-SES schools is students’ own low-SES family background.

Our study has two main limitations. The first is that PISA data are cross-sectional, which means that causality cannot be inferred. The second is that our analytical strategy—namely the quantile regression approach—does not allow us to use multilevel models with PISA’s complex sampling structure. We have addressed this limitation by estimating standard errors clustered at the school level; please refer to endnote three for more detail about our approach. Despite these limitations, our study provides novel insights that we believe make a strong contribution to the literature.

Based on our findings, we recommend two future lines of research. The first line of research could examine whether our findings are replicable and generalisable, to different national contexts, age groups and measures of academic achievement. If other studies show similar findings, then we will be able to say with some certainty that school SES effects do not vary widely by student achievement level but are relatively more impactful for lower performing youth. On the other hand, if studies show dissimilar findings, then there will be an opportunity to develop a rich and nuanced theoretical framework about the factors that explain these differential effects. For example, it is plausible that school SES effects are minimal for some or even all students when school segregation is not pronounced, as found by Stewart et al. (2019) in their study of pre-school settings in the UK, or Jehangir et al. (2015) for Finland. Similarly, it is plausible that school SES effects do not vary by achievement level in national contexts with a small proportion of private, fee-charging schools. These are just some examples of hypotheses that could be tested for the purpose of expanding our theoretical understanding of school SES effects.

The second line of future research that we recommend concerns the importance of schooling for students from different social backgrounds. In particular, we recommend that more research be conducted about the differential effects of school SES on achievement for the purpose of deepening our theoretical understanding of the impacts of school socioeconomic composition, and school quality and effectiveness more generally, on students. Whether schooling “matters” more for disadvantaged students than it does for their more advantaged peers is an open question in the literature. On the one hand, schooling might be more important for disadvantaged students because they enjoy fewer supports and resources outside school that can foster the formal academic learning that occurs in schools (Portes, 2005; Sewell & Hauser, 1980; Turkheimer et al., 2003). On the other hand, schooling might be more impactful for advantaged students because the extensive informal learning opportunities they receive outside school provide a strong foundation for maximising learning opportunities provided in school. In this “it takes money to make money” analogy, students who enjoy extensive supports and resources outside school are better able to leverage the supports and resources in school. Finally, these trajectories are not necessarily mutually exclusive—it could be the case that schooling matters the most for students from *both* the most advantaged and disadvantaged backgrounds, with the relationship between schooling and outcomes representing a “U” shape rather than a diagonal line. Or all these trajectories can amplify and cancel each other out, for a variety of reasons that may vary by student group, with the result that schooling matters the same for all students, but for different reasons. Clearly there is much scope for theory building and hypothesis testing about the differential impacts of schooling generally, and school social composition specifically, on student outcomes.

Our findings also have implications for policymaking. As the positive association of school SES with academic achievement is similar for all students regardless of their individual performance level, policies that promote school social segregation should not be pursued based on the erroneous assumption that they only benefit some students. Moreover, school socioeconomic segregation is problematic because it compounds the multiple educational inequalities already faced by socially disadvantaged students and schools, without necessarily increasing the achievement of students in socially advantaged schools or the overall achievement of students at the societal level (Gorard & Siddiqui, 2018; OECD, 2016). In other words, school social segregation is neither equitable nor efficient. Policies and structures that promote school segregation should therefore be avoided. This is especially important since it is plausible that the greater the degree of school segregation, the greater the degree of educational inequalities. While school social segregation exists to some extent in all countries, cross-national data from OECD countries show that the degree to which schools are socially segregated varies widely. While the reasons for these cross-national variations are undoubtedly multiple and complex, educational structures and policies that exacerbate school social segregation, such as those related to educational marketization and its underlying dynamics of choice and competition (Alegre & Ferrer, 2010; Bonal & Bellie, 2018), should be avoided or at least mitigated. Finally, we note that it is much more feasible for education policy makers to reduce school socioeconomic composition effects, by reducing school socioeconomic segregation, than it is to reduce the effects of family-level socioeconomic status via the reduction of poverty and income inequality.

School socioeconomic segregation appears to be both a driver and manifestation of socioeconomic stratification in education. It contributes to reproducing the educational disadvantages that socioeconomically differentiated performance reflects. Australia is unlikely to break the intergenerational perpetuation of SES-linked school outcomes, to prepare youth for citizenship in a democratic and just multi-ethnic society, or to equip every child to fully participate in a globalizing high-tech economy if we do not again consider the socioeconomic composition of the schools we provide for our children.

## Data Availability

The data analysed in this study are publicly available from the Australian Council for Educational Research at https://www.acer.org/au/pisa/publications-and-data.

## Footnotes

1 New South Wales, which enrols approximately 30% of all students in Australia, is the only state that has a sizeable number of fully and partially selective secondary schools. In 2019, approximately 4% of students in NSW attended a selective school (based on author calculations from data from ACARA (https://www.acara.edu.au/reporting/national-report-on-schooling-in-australia/national-report-on-schooling-in-australia-data-portal/student-numbers#View2) and NSW Department of Education (https://education.nsw.gov.au/public-schools/selective-high-schools-and-opportunity-classes/year-7/what-are-selective-high-schools/places-available-in-selective-high-schools); sites accessed April 6, 2021).

2 Choice is promoted through the public (federal and state) subsidies to private schools. These subsidies go directly to schools; they are not a tuition voucher provided to families. Public funding of private schools has enhanced learning facilities and supports at these schools, not reduced tuition fees (Watson & Ryan, 2010).

3 According to Marsh et al. (2009), there are two basic issues to consider in creating a school-level measure from student-level values. The first issue is whether the aggregation involves a sampling of items from a broader pool of items pertaining to the construct involved. For instance, a latent school climate measure composed of 10 manifest items may be subject to measurement error if the 10 items are a subset of a potentially larger pool of items addressing school climate traits beyond those that the 10 items address. If the aggregation does not suffer from such an undermeasurement problem or if the degree of the problem is tolerable, then a direct average of the manifest items is acceptable in crafting a higher-level composite. This was the case in our study, allowing us to specify school SES as a direct mean of student SES, using student sampling weights in the process. PISA’s SES measure is a pre-calculated weighted average of three indices: parental educational attainment (in years), parental occupational status on the “International Socio-Economic Index” (ISEI) scale (Ganzeboom, 2010; Ganzeboom et al., 1992), and a measure of an exhaustive set of “household possessions” (as a robust marker of income and wealth). These three components account for financial, social, cultural, and human capital resources available to the student, a well-established approach that sufficiently captures all fundamental components of SES with minimal measurement error (Cowan et al., 2012; Ensminger & Fothergill, 2003; Sirin, 2005; Willms & Tramonte 2019). According to Avvisati (2020), this approach can be improved to a degree by incorporating building-level values into the school SES composite, such as whether the school has a “Title I” status (a designator used in the U.S.) and the percentage of students eligible for lunch subsidy. However, such administrative measures are not uniformly available in PISA data. The second issue Marsh et al. (2009) raise is about sampling error at the school level. Here, the question is whether the sample of students nested in a school is representative of the building. When the risk of sampling error is high, the ideal approach is to utilize latent aggregation. This can be accomplished by specifying the school-level mean as a level-2 latent variable in a multilevel regression context (Becker et al., 2018). This approach was infeasible in our case because a multilevel quintile regression procedure—especially one involving 10 plausible values of the outcome measure along with 80 replicate BRR (“balanced repeated replication”) weights, and 0.5 Fay adjustment—is beyond current technical capabilities. However, we are confident that potential sampling error at the school-level in PISA data does not introduce an intolerable degree of bias in our findings. This is likely because of PISA’s high quality sampling design. Students are sampled with *equal probability* within schools, meaning each re-sampling of students from a building would yield a similar batch of students as before from the building (OECD, 2018a, 2018b). This student-level procedure is combined with “probability-proportional-to-size-sampling” at the building level, which ensures school-level representation nationally (see Skinner, 1988). Students in PISA data receive a final weight which indicates how many other students from the population are represented. The final student weight involves *both* the within-school student weight (the inverse of the student’s probability of selection) and the school weight itself (the inverse of the school’s probability of selection). Given the equal probability sampling approach within schools and the components of the final student weight, manifest aggregation of student SES values creates robust school-level SES values that are subject to limited sampling error. Thus, while we are unable to entirely eliminate within-school sampling error, we believe the problem does not severely undermine our analysis. Ultimately, our approach to creating school-level SES from student-level values is similar to that commonly used in the field, as noted by Willms (2010).

4 As the proportion of Indigenous students is small (less than 3%), we tested whether the coefficient sizes changed when they were removed from the sample and found no meaningful differences. Methodologically, it is important to keep Indigenous students in the sample as the data are sample-weighted. Conceptually, it is important to keep them in the sample so that the Australian reality is accurately reflected.

5 We employed “error propagation” rules for calculating the standard error for a ratio of two estimates where each has its own standard error. We also drew on insights from Dunlap and Silver (1986). In summary, the standard error for an SHIR estimate (SE_SHIR_) is given by: SE_SHIR_ = Sqrt[(SE_SCSES_/SCSES)^2^ + (SE_STSES_/STSES)^2^]*SHIR. where SE_SCSES_ and SE_STSES_ are the standard errors of the regression coefficients for school and student SES, respectively, and SCSES and STSES are the school and student SES coefficients themselves. Applying the above formula to the light shaded bars in Fig. 3 involved an additional *prior* step where we had to calculate the standard error of the total effect of SCSES, an effect comprised of two parts: the main effect (“β_m_”) and the interaction effect (“β_i_”). In such situations involving two coefficients, the standard error of the total effect is given by: Sqrt[Var(β_m_) + Var(β_i_) + (2*Cov(β_m_,β_i_))]. where Var(β_m_) and Var(β_i_) are the variances of the main and interaction effects respectively, and Cov(β_m_,β_i_) is the covariance of the two coefficients. These are obtained from the variance–covariance matrix following each relevant multivariate estimation model in Tables 1–3.

6 Confidence interval for student SES estimate is 0.159 ± (1.96*0.014) = [0.132, 0.186]. Confidence interval for school SES estimate is 0.250 ± (1.96*0.030) = [0.191, 0.309].
